# Maternal health during pregnancy and oral health of 4-year-olds: a birth cohort study from Brazil

**DOI:** 10.1590/1807-3107bor-2025.vol39.038

**Published:** 2025-04-04

**Authors:** Clarissa Nachtigall FÔLHA, Helena Silveira SCHUCH, Sara Arangurem KARAM, Marlos Rodrigues DOMINGUES, Pedro Curi HALLAL, Flávio Fernando DEMARCO

**Affiliations:** (a) Universidade Federal de Pelotas – UFPel, School of Dentistry, Pelotas, RS, Brazil.; (b) Universidade Católica de Pelotas – UCPel, Graduate Program in Health in the Vital Cycle, Pelotas, RS, Brazil.; (c) Universidade Federal de Pelotas – UFPel, School of Physical Education, Graduate Program in Physical Education, Pelotas, RS, Brazil.; (d) University of Illinois Urbana, Department of Kinesiology and Community Health, Champaign, IL, USA.

**Keywords:** Exercise, Maternal Health, Dental Caries, Child, Preschool, Oral Health, Cohort Studies

## Abstract

Data were collected by trained interviewers at primary healthcare units and hospitals during pregnancy and childbirth, and by trained dentists when the children were 4 years old. A total of 3,644 mothers and 3,645 babies were included in the study, which evaluated the association between systemic diseases, maternal physical activity, weight gain during pregnancy, and the oral health of four-year-old children enrolled in the 2015 Pelotas Birth Cohort (Brazil). Exposure variables included systemic diseases, maternal physical activity, and gestational weight gain. Outcome variables were dental caries and caries experience in four-year-old children, assessed using the ICDAS index. Statistical analyses were performed using the Stata 15 software, and they included Pearson’s chi-square test and Poisson regression to estimate prevalence ratios and their respective 95% confidence intervals. Excessive weight gain during pregnancy was associated with a higher risk of dental caries in children (PR: 1.12; 95%CI: 1.01–1.23). Maternal physical activity before or during pregnancy was considered a protective effect against dental caries in the unadjusted analysis ([RP: 0.82; 95%CI: 0.71–0.93] [RP: 0.60; 95%CI 0.44–0.81]), respectively, but this association was no longer significant after adjusting for confounding factors. The presence of maternal systemic diseases during pregnancy was not associated with childhood caries. In conclusion, maternal systemic diseases during pregnancy were not associated with dental caries in children. On the other hand, an excessive increase in maternal weight during pregnancy was associated with poorer oral health among children.

## Introduction

Dental caries is a global health concern, especially among children.^1^ According to the World Health Organization, it is the most important oral health condition, considering that children with caries in their primary teeth are at a greater risk of developing caries and having associated complications in adulthood.^2^ A meta-analysis found that the global prevalence of dental caries in primary teeth was 46.2% between 1995 and 2019.^3^ In Brazil, according to data from the most recent oral health survey, the prevalence of dental caries was 53.4% in children aged 5 years.^4^ The prevalence of untreated dental caries was 45.5% among five-year-old children in Pelotas, southern Brazil, according to the 2004 Pelotas Birth Cohort.^5^

In the attempt to prevent dental caries and consequently reduce its prevalence during childhood, pregnancy can be an ideal period for oral health promotion because of the influence of maternal health behaviors on children’s oral health status.^6^ A systematic review showed a reduction in the incidence of childhood caries when mothers received prenatal oral health care, acknowledging that such care is a protective factor in the first four years of a child’s life.^7^

Some maternal factors are associated with the oral health of children, as evidenced by a birth cohort in southern Brazil, in which poor maternal oral health (higher incidence of dental caries) was a risk factor for a higher prevalence of dental caries among children.^8^ Furthermore, mothers who do not visit the dentist regularly tend to postpone their children’s dental appointments.^9^ The leading cause of dental caries is frequent sugar intake, and caregivers are directly responsible for their children’s diet during childhood.^10^ A recent systematic review concluded that higher sucrose consumption, poor toothbrushing, and lack of oral hygiene guidance from caregivers were associated with childhood caries.^11^However, maternal oral health or hygiene is just one of the many factors that influence children’s caries. Overall maternal health also impacts children’s oral health. A study demonstrated that the relative risk of developing dental caries among three-year-old children whose mothers were younger, smoked during pregnancy, had a low family income, and/or lived in countries with high rates of poverty and obesity was five times higher when compared to children born to mothers without these risk factors.^12^ Pregnancy is a critical time for mothers to establish habits that can, in turn, shape the habits of their children.

Physical activity during pregnancy can be seen as both an indicator of maternal health and a predictor of children’s oral health in the future, and it should therefore be encouraged.^13^ Physical activity also appears to be associated with cognitive and language development by the age of 4 years.^14^ Maternal obesity during pregnancy can increase the risk of childhood obesity,^15^ and there is evidence of an association between childhood overweight/obesity and dental caries.^16^ On the other hand, systemic diseases during pregnancy may hinder physical activity and increase the risk of gestational obesity.^17^ Although some studies have investigated the effects of habits on children’s oral health, studies investigating the effect of systemic diseases, physical activity, and weight gain during pregnancy on children’s oral health are still lacking. The aim of this study was to evaluate the potential impact of systemic diseases, maternal physical activity, and gestational weight gain on the oral health of 4-year-old children in a birth cohort from southern Brazil.

## Methods

This study followed the Strobe guidelines for longitudinal studies.^18^

Ethical aspects: The study protocol was developed and approved by the Federal Council Research Ethics Committee of the University of Pelotas (nº 26746414.50000.5313 and 717.271/2014).

Pelotas is a town in southern Brazil with approximately 350,000 inhabitants. The 2015 cohort recruited pregnant women during prenatal care with prospective collection of pregnancy-related variables. Additionally, a parallel prenatal clinical study involved 73.8% of the mothers who subsequently gave birth to children included in the cohort. All children born in hospitals between January 1 and December 31, 2015 were eligible for inclusion if their mothers lived in the urban area of Pelotas. A small fishing village, no longer classified as urban, and an isolated urban area that was later annexed by the neighboring town of Capão do Leão were included in the pre-2015 cohorts. Births from these two areas were included in the cohort to maintain the comparability with studies from these previous cohorts. Research teams were deployed daily to the four hospitals where 99.9% of all births occurred. A fifth hospital was visited daily by a mobile team. Most mothers of eligible children were interviewed within 1 day (96.7% of interviews) or within 2 days after delivery (99.2%).^19^

### Prenatal care

The 123 health units and private clinics providing prenatal care in the municipality were visited or contacted weekly between May 2014 and December 2015 to identify pregnant women expecting to give birth during 2015. These women were either visited at home or invited to participate in the clinical survey between 16 and 24 weeks of gestation to answer a health questionnaire and receive an accelerometer. During prenatal care, 10 individuals (interviewers) underwent theoretical and practical training to standardize the data collection process. The interviews were scheduled according to gestational age, aiming for at least one interview during pregnancy. Pregnant women interviewed before 16 weeks of gestation completed the ‘initial contact’ questionnaire, and a second interview was subsequently conducted from the 16th to the 24th weeks, focusing mainly on the mother’s lifestyle habits.^19^ Women enrolled after 16 weeks of gestation answered a combined assessment questionnaire that integrated both the baseline and main assessment questionnaires.

### Perinatal care

Throughout 2015, all live births and stillbirths that occurred in the five hospitals were identified. Mothers of live births were invited to participate in the cohort. After signing the informed consent form, the mothers were interviewed about their health and behaviors during pregnancy and childbirth. Of a total of 4,333 live births eligible for the 2015 cohort, 4,275 (98.7%) were included in the study in the perinatal period after considering losses to follow-up and refusals to participate.^19^

### 48-month follow-up of the children

At 48 months, the assessments were performed at the Epidemiological Research Center, where clinical evaluations were conducted and questionnaires were applied using REDcap software. The clinical oral examination was conducted by trained and calibrated dentists. In the present study, the mean inter-examiner kappa agreement for dental caries was 0.9076 (0.8339–0.9568). Early childhood dental caries was the outcome of interest, assessed using the International Caries Detection and Assessment System (ICDAS). The merged ICDAS classification categorized tooth surfaces for the presence of dental caries into four categories: 0, no evidence of caries; A, initial caries; B, moderate caries; and C, extensive caries. The ICDAS also separately classified tooth surfaces for the presence of dental restorations. Three additional codes were included in both assessments (dental caries and restorations): 97, missing surface due to dental caries; 98, missing surface due to other reasons; and 99, unerupted tooth surface. The merged ICDAS classification follows a protocol for the diagnosis of early childhood caries developed by researchers from the World Health Organization (ECC-0 sound; ECC-1 smooth white spot lesion; ECC-2 enamel breakdown; and ECC-3 dentin caries). For the analysis, the dmfs index was derived from the ICDAS as follows: Decayed surface (d) included moderate caries and extensive caries (ECC-2 and ECC-3), excluding initial enamel lesions as they are not part of the dmfs index; missing surface (m) included missing surface due to dental caries; and filled surface (f). The dmfs index was incorporated into analytical models in two ways: first as the presence of ECC (no [dmfs = 0] and yes [dmfs ≥ 1]) and second as the severity of ECC (number of decayed, missing, and filled tooth surfaces).^20^

### Exposure variables

Exposure variables included maternal systemic diseases, gestational weight gain, and maternal physical activity, before pregnancy and in the prenatal period. All variables were collected in the prenatal and/or perinatal period.

### Maternal systemic diseases

Information on general health was gathered in the prenatal period, including highly prevalent diseases such as hypertension, diabetes, frequent bleeding, heart disease, and any physical disability that restricted physical activity. The responses to the questions were dichotomized into “no disease” when pregnant women said they did not have any of these diseases and “at least one disease” when they reported having hypertension and/or diabetes and/or frequent bleeding and/or heart disease and/or physical disability.

### Gestational weight gain

Maternal weight at the beginning of pregnancy was obtained from prenatal records or reported by the mother at the time of birth. Pre-pregnancy BMI was calculated by dividing the mothers’ weight by their height squared.^19^ To calculate gestational weight gain, mothers were asked during a perinatal interview about their weight at the end of pregnancy and before delivery.^21^ Total gestational weight gain was then calculated by subtracting weight at the end of pregnancy from pre-pregnancy weight. The WHO criteria were applied to classify gestational weight gain in relation to pre-gestational BMI, according to guidelines from the Brazilian Ministry of Health^22^for prenatal care. According to the guidelines, underweight women should gain between 12.5 and 18 kg during pregnancy, normal-weight women between 11.5 and 16 kg, overweight women between 7 and 11.5 kg, and obese women between 5 and 9 kg. Total gestational weight gain was categorized as “insufficient”, “adequate,” or “excessive” based on the established range of weight gain.^23^

### Physical activity

Physical activity patterns were determined in the prenatal period through questions related to physical exercise performed by pregnant women before and during pregnancy. The answers were dichotomized into “no” (does not engage in physical activity) and “yes” (engages in physical activity), regardless of frequency and/or duration, both in the pre-pregnancy period and during pregnancy. Physical activity data were obtained in the second trimester of pregnancy.

### Covariates

The following covariates were collected in the prenatal and perinatal periods: maternal age (collected in completed years and categorized as < 20, 20–34, and ≥ 35 years); self-reported maternal race/color (collected according to the classification of the Brazilian Institute of Geography and Statistics: white, black, Asian, mixed race, or indigenous); maternal education (collected in completed years of schooling and categorized into 0–4, 5–8, 9–11, and ≥ 12 years of schooling); family income (collected in reais and categorized into quintiles); and sugar consumption trajectory (always low; always intermediate; growing; and always high). Sugar consumption trajectory was modeled as a variable based on sugar intake data at the 3-, 12-, 24-, and 48-month follow-up periods, using group-based trajectory modeling. Children with valid answers in three or more follow-up visits were included in the construction of sugar consumption trajectories. The logit distribution was adopted, considering the dichotomous distribution of sugar consumption at each time point.^24^

### Outcome variables

The outcomes of interest were dental caries and caries experience in children examined at 48 months of age. These outcomes were evaluated using the simplified ICDAS (Simplified International Caries Detection and Assessment System) index. For the dental caries outcome, the presence of moderate caries and/or advanced carious lesions was considered. Caries experience was defined by the presence of a tooth surface affected by early-onset to advanced-stage caries, or a restored surface or missing surface resulting from caries. Both outcomes were categorized dichotomously (no or yes), where “yes” indicated the presence of the outcome of interest.

### Statistical analyses

Statistical analyses were performed using Stata version 15. Descriptive analyses of all variables were conducted using absolute values and relative frequencies, whereas bivariate analyses of covariates for caries experience and untreated dental caries were performed using Pearson’s chi-square test. Prevalence ratios and their respective 95% confidence intervals were estimated using Poisson regression with robust variance for each exposure-outcome association. Unadjusted and adjusted estimates were evaluated for each association.

## Results

A total of 4,328 mothers were evaluated during pregnancy in the original 2015 Pelotas (Brazil) Cohort study. In our study, 84.2% of mothers (n = 3,644) were included, and our analytical sample was similar to the total cohort sample (Table 1). Women were excluded from our study for reasons such as: stillbirth or ineligibility at the time of delivery. Most women were aged 20 to 34 years (71% [n = 2,589]), were white (69.9% [n = 2,546]) and had completed between 9 and 11 years of education (35.2% [n = 2,546]). = 1,283]) (Table 2). Most of the children assessed at 4 years of age showed increasing sugar consumption, 34.4% (1,271), followed by intermediate consumption, 32.2% (1,175) (Table2). Regarding the presence of at least one systemic disease, 24.2% (n = 673) of pregnant women were diagnosed with at least one gestational condition. Regarding gestational weight gain, 35.7% (n = 1,254) of pregnant women had excessive weight gain and 29.8% (n = 1,045) had insufficient weight gain during pregnancy. Around 40% (39.8% [n = 1,098]) of the women in the sample reported performing physical activity before pregnancy, but the frequency of physical activity was 9% (n = 254) during pregnancy. The oral health examination at 48 months of age revealed a prevalence of dental caries and untreated dental caries of 37.4% (n = 1,362 [95%CI 35.8–38.9]) and 26% (n = 947 [95%CI 24.6–27.4]), respectively (Table 2). Table 3 presents the prevalence of oral health status according to maternal health and physical activity. Among mothers who practiced physical activity during pregnancy, 15.4% (95%CI 11.4–20.3) had children with dental caries at 4 years of age and 27.2% (95%CI 22.0–32, 9) had children with caries experience. On the other hand, 25.5% (95%CI 23.8–27.2) of untreated caries and 36.6% (95%CI 34.7–38.4) of caries experience were found among those who did not practice physical activity during pregnancy. When we analyzed the weight gain of pregnant women, we found that those with excessive weight gain during pregnancy had children with a higher prevalence of untreated caries (27.1% [95%CI 24.7–29.6]) and greater caries experience (38.8% [95%CI 36.1–41.5]) compared to children of women who gained adequate and/or insufficient weight. Regarding the general health status of pregnant women, those diagnosed with at least one systemic disease had children with a higher prevalence of untreated caries and caries experience, 27.0% (95%CI 23.8–30.5) and 39.2% (95%CI 35.6–42.9), respectively (Table 3). Table 4 shows the unadjusted and adjusted analyses of the associations between variations in exposure and the outcome. After adjustment for age, skin color, income, and education, the prevalence of untreated caries was 18% higher (PR 1.18 [95%CI 1.03–1.35]) among children of mothers who gained excessive weight during pregnancy and the prevalence of caries experience was 12% higher (PR 1.12 [95%CI 1.01–1.24]). Although physical activity before pregnancy had a protective effect against caries experience in the unadjusted analysis, this effect was no longer significant in the adjusted analysis. The same pattern was found for physical activity during pregnancy. The presence of systemic diseases during pregnancy was not associated with the prevalence of caries in children, both in the unadjusted and adjusted analyses.

**Table 1:** Mothers of the 2015 Pelotas Birth Cohort.

Perinatal characteristics	Original sample	Oral health sample	
	n (%)	n (%)	p-value*
Maternal age			0.912
< 20	632 (14.6)	525 (14.4)	
20–34	3,056 (70.6)	2,589 (71.1)	
≥ 35	640 (14.8)	530 (14.5)	
Total	4328 (100)	3,644 (100)	
Maternal skin color			0.840
White	3,050 (70.6)	2,546 (70.0)	
Black/Brown	1,246 (28.8)	1,071 (29.4)	
Other	26 (0.6)	22 (0.6)	
Total	4,322 (100)	3,639 (100)	
Maternal schooling			0.677
0–4	399 (9.2)	315 (8.6)	
5–8	1,109 (25.6)	936 (25.7)	
9–11	1,479 (34.2)	1,283 (35.2)	
≥12	1,341 (31.0)	1,110 (30.5)	
Total	4,328 (100)	3,644 (100)	
Family income in BRL (quintiles)			0.763
1	866 (20.0)	710 (19.5)	
2	866 (20.0)	748 (20.5)	
3	865 (20.0)	752 (20.6)	
4	866 (20.0)	738 (20.3)	
5	865 (20.0)	695 (19.1)	
Total	4,327 (100)	3,643 (100)	

**Table 2:** Description of gestational/perinatal mother-child characteristics and at 48 months.

Variable	n	%
Prenatal period		
Systemic disease (n = 2,779)		
No disease	2,106	75.8
At least one disease	673	24.2
Gestational weight gain (n = 3,505)		
Insufficient	1,045	29.8
Adequate	1,206	34.4
Excessive	1,254	35.8
Pre-pregnancy physical activity (n = 2,757)		
No	1,659	60.2
Yes	1,098	39.8
Gestational physical activity (n = 2,809)		
No	2,555	91
Yes	254	9.0
Perinatal period		
Maternal age (n = 3,644)		
< 20	525	14.4
20–34	2,589	71.1
≥ 35	530	14.5
Maternal skin color (n = 3,639)		
White	2,546	70.0
Black/Brown	1,071	29.4
Other	22	0.6
Maternal schooling (n = 3,644)		
0–4	315	8.6
5–8	936	25.7
9–11	1,283	35.2
≥ 12	1,110	30.5
Family income in BRL (quintiles) (n = 3,643)		
1	710	19.5
2	748	20.5
3	752	20.6
4	738	20.3
5	695	19.1
Follow-up at 48 months – Child’s Oral Health		
Sugar consumption trajectory (n = 3,645)		
Always low	874	24.0
Always intermediate	1,175	32.2
Growing	1,271	34.9
Always high	325	8.9
Caries experience (n = 3,645)		
No	2,283	62.6
Yes	1,362	37.4
Dental carie (n = 3,645)		
No	2,698	74.0
Yes	947	26.0

**Table 3:** Distribution of outcomes according to main exposures.

Variable	Caries experience		Dental carie	
	No	Yes	No	Yes
	% (95%CI)	% (95%CI)	% (95%CI)	% (95%CI)
Pre-pregnancy physical activity				
No	63.4 (61.0–65.6)	36.6 (34.4–38.9)	73.6 (71.4–75.6)	26.4 (24.3–28.6)
Yes	65.7 (62.8–68.4)	34.3 (31.6–37.2)	78.4 (75.9–80.8)	21.6 (19.2–24.1)
Gestational physical activity				
No	63.4 (61.6–65.3)	36.6 (34.7–38.4)	74.5 (72.8–76.2)	25.5 (23.8–27.2)
Yes	72.8 (67.0–77.9)	27.2 (22.0–32.9)	84.6 (79.7–88.6)	15.4 (11.4–20.3)
Gestational weight gain				
Insufficient	62.8 (59.7–65.6)	37.3 (34.4–40.3)	73.7 (70.9–76.3)	26.3 (23.7–29.1)
Adequate	65.3 (62.6–67.9)	34.7 (32.0–37.4)	76.9 (74.5–79.2)	23.1 (20.7–25.5)
Excessive	61.2 (58.5–63.9)	38.8 (36.1–41.5)	72.9 (70.4–75.3)	27.1 (24.7–29.6)
Systemic diseases				
No disease	65.5 (63.4–67.5)	34.5 (32.5–36.6)	76.2 (74.3–77.9)	23.7 (22.0–25.6)
At least one disease	60.8 (57.0–64.4)	39.2 (35.6–42.9)	73.0 (69.5–76.2)	27.0 (23.8–30.5)

95%CI: 95% confidence interval.

**Table 4:** Association between maternal gestational health and dental caries at 4 years old. 2015 Pelotas Birth Cohort.

Variable	Caries experience (Yes)		Dental carie (Yes)	
	PR (95%CI)^a^	PR (95%CI)^b^	PR (95%CI)^a^	PR (95%CI)^b^
Pre-pregnancy physical activity				
No	Ref.	Ref.	Ref.	Ref.
Yes	0.94 (0.84–1.04)	1.08 (0.98–1.20)	0.82 (0.71–0.93)	0.99 (0.87–1.14)
Gestational physical activity				
No	Ref.	Ref.	Ref.	Ref.
Yes	0.74 (0.60–0.91)	0.92 (0.75–1.14)	0.60 (0.44–0.81)	0.81 (0.60–1.09)
Gestational weight gain				
Adequate	Ref.	Ref.	Ref.	Ref.
Insufficient	1.08 (0.96–1.20)	1.02 (0.92–1.14)	1.14 (0.98–1.31)	1.07 (0.93–1.24)
Excessive	1.12 (1.01–1.24)	1.12 (1.01–1.24)	1.18 (1.03–1.34)	1.18 (1.03–1.35)
Systemic diseases				
No disease	Ref.	Ref.	Ref.	Ref.
At least one disease	1.14 (1.02–1.26)	1.08 (0.97–1.21)	1.14 (0.98–1.31)	1.06 (0.92–1.22)

PR: prevalence ratio; 95%CI: 95% confidence interval. ^a^Crude analysis; ^b^Analysis adjusted for age, skin color, income, schooling and sugar consumption trajectory.

## DIscussion

Our study showed that the presence of systemic diseases during pregnancy was not associated with the analyzed outcomes. Although physical activity before or during pregnancy played a protective role in the prevalence of dental caries in children at the age of 4 years, this effect disappeared after adjustments. Conversely, excessive maternal weight gain during pregnancy was associated with the prevalence of dental caries and caries experience in their children by the age of 4 years.

Few studies in the literature examine the association between systemic diseases, physical activity, and weight gain in pregnant women, and their relationship with the oral health of their children. Furthermore, knowledge about associations between lifestyle during pregnancy and the development of dental caries in childhood is limited.^25^ Maternal behaviors can influence the severity of dental caries during childhood. A cohort study of young mothers from Pelotas showed that behaviors such as living with a partner, having a positive perception of the child’s oral health and looking at their children’s teeth and mouth were associated with the occurrence of childhood caries.^26^ Moreover, mothers with a higher prevalence of caries had children with more carious lesions.^8^

Education and health promotion should begin as early as possible, even during pregnancy, to prevent children from developing oral diseases that could require therapeutic treatment and generate negative impacts for them, their families, and society. In fact, nutritional counseling programs for mothers appear to have a protective effect on the occurrence of early childhood caries.^27^ In addition to dentists, other health professionals, such as pediatricians, nurses, obstetricians, and family doctors should be encouraged to engage in education and counseling initiatives to prevent the occurrence of dental caries. These professionals generally interact with mother and child more frequently than do dentists. Involving these professionals in collaborative care with oral health professionals and delegating health care areas to the multidisciplinary team could provide better preventive outcomes.^28^ Moreover, the gestational period seems to be an appropriate time for the health systems to invest in public health promotion policies, given that pregnant women have frequent contact with health teams during this period, allowing for more effective delivery of health educational guidance aimed at preventing potential risks for both general and oral health. Previous studies have already shown that health education provided to mothers during pregnancy would produce beneficial effects on their children.^29^

Caries and obesity share common risk factors, including excessive sucrose intake and socioeconomic status,^30^ but frequent and/or excessive consumption alone does not fully account for the development of obesity. Excessive weight gains during pregnancy, leading to overweight and obesity, may be linked to unhealthy maternal eating habits. These habits could perhaps be passed on to their children, potentially increasing the prevalence of dental caries, as observed in our study. Uerlich et al.^30^studied common determinants of dental caries and obesity in children from a birth cohort in the United Kingdom and found that caregivers’ eating habits were associated with the prevalence of caries. Caregivers with an unhealthy diet can instill similar patterns in their children.^30^ According to our findings, maternal obesity was found to increase the risk of caries in children evaluated, as evidenced in a longitudinal study.^12^ Factors such as obesity and lower maternal education were associated with the risk of experiencing dental caries at the age of 5 years.^25^ Akinkugbel et al.^31^found an association between pre-pregnancy BMI, weight gain, and childhood caries and suggested that it could be due to pre-pregnancy factors. BMI is a more distal risk factor compared to childhood obesity. Those authors further suggested that the relationship between maternal BMI and childhood caries is complex because it would be necessary to determine whether the risk of dental caries arises from difficult biological influences on the child, transfer of eating habits, confounding factors including social, environmental, and other determinants. While sugar has been associated with obesity, we did not measure sugar intake during pregnancy, but we evaluated the trajectory of children’s sugar intake during the first 48 months and found an association with dental caries in children.^24^ We cannot rule out the possibility that excessive weight gain during pregnancy was due to the consumption of foods with high sugar content. Mothers with a higher intake of sugar would be more likely to have children ingesting more sugar and consequently having more risk of dental caries.

Perhaps, the relationship involving obesity, eating habits, and physical activity represents a general health concern among women, which is not easily measured as distinct factors, but such relationship may contribute to better oral health outcomes. Furthermore, physical activity can act as a marker of health, with physically active people tending to have healthier diets, although we do not yet know to what extent this association is explained biologically or from a behavioral point of view.^32^ Medapati & Pachava^33^carried out a systematic review and studied the effect of physical activity on oral health and concluded that individuals who practice physical activity regularly have a decrease in inflammatory biomarkers, which results in an improvement in periodontal disease. Furthermore, they found an association between oral health and oral health behaviors (oral hygiene practices) among individuals who practiced physical activity regularly. However, in our study, the assessment of maternal physical activity did not influence the oral health of their children. In our sample, most pregnant women did not practice physical activity before (60.2%) and during (90.1%) pregnancy. Physical activity during pregnancy, as in other periods of life, is influenced by people’s socioeconomic status and educational level. Generally, richer or more educated individuals tend to practice more physical activities.^34^ It is also important to highlight that physical activity tends to decrease as pregnancy progresses.^37^ For Tornquist et al.,^35^motherhood is associated with a reduction in leisure-time physical activity among women. The prevalence of active mothers decreases in the prenatal and postpartum periods compared to the preconception period. These levels only increase twelve months after giving birth. In our study, physical activity, both before and during pregnancy, was not associated with children’s oral health after adjustments, but this practice should be encouraged in pregnant women due to the benefits it brings to the general health of the mother and fetus. Practicing physical activity during pregnancy can stimulate the metabolism of mesenchymal stem cells in babies.^36^ Ricardo et al.^37^found that children of mothers who exercised during pregnancy were more active in their first years of life. Dipietro et al.^38^found that moderate physical exercise during pregnancy reduces the risk of excessive weight gain during pregnancy, as well as symptoms of diabetes and depression in the postpartum period. Children born to mothers with obesity and who followed a diet rich in fat and sugar during pregnancy had a higher prevalence of dental caries at the age of 5 years.^25^ The greater intake of fatty and sugar-rich foods leads to the occurrence of systemic diseases such as hypertension, diabetes, and cardiovascular diseases, whether during pregnancy or not, and these mothers tend to reproduce their diets in their children who, in turn, develop caries in their first years of life.^21^

It has been demonstrated that dental caries is a chronic disease strongly influenced by socioeconomic factors, with most of the disease burden occurring in the more economically vulnerable populations, as shown by national oral health surveys.^4^ Furthermore, in Brazil, skin color is a strong socioeconomic marker, as well as educational level. The occurrence of dental caries is higher among children whose mothers are less educated.^8^ More educated individuals tend to have more knowledge about health/disease processes, tending to have healthier habits and more frequent access to health care , presenting a better general health status and better oral health indicators. Regarding maternal age, older mothers are less likely to have dental caries compared to younger mothers. A likely reason for this conclusion is that older mothers tend to have higher levels of education and greater accumulation of social capital, which could influence the establishment of healthier habits in their children.^11^ Furthermore, older mothers are generally more mature and take more care of their children.^30^

The strength of this study lies in its longitudinal design. Another highlight is the availability of data on maternal health in the gestational and perinatal periods, allowing for the association between physical activity, weight gain, and maternal systemic diseases. Furthermore, the availability of data on the assessment of children’s clinical oral examination is another strength of this study. Finally, we had access to several mother and child covariates that allowed us to control for relevant confounders, thereby minimizing potential bias.

A limitation of our study may be the fact that the practice of physical activity, weight gain, and the presence of maternal systemic diseases are more distal determinants for dental caries and caries experience during childhood. Furthermore, not all mothers of children in the 2015 cohort were monitored during prenatal care and, therefore, were not included in the present study.

## Conclusion

The findings of this study demonstrate that children of mothers who had excessive weight gain during pregnancy had a higher prevalence of dental caries and caries experience at 4 years of age. Although maternal physical activity is not associated with dental caries among the children in our study, pregnant women practicing physical activity could encourage them to have healthier habits, reducing the risk of gestational obesity, in addition to having a positive impact on their children’s overall health. Furthermore, the gestational period is a critical time for health systems to invest in public health promotion policies, given that it is during this period that pregnant women have greater contact with health teams and should receive greater educational and preventive support for pregnancy for their general and oral health.
